# Dietary Nutrition, Gut Microbiota, and Health Status Across Geographically Diverse Populations in Mongolia: A Cross‐Sectional Study

**DOI:** 10.1002/fsn3.70531

**Published:** 2025-07-04

**Authors:** Zhixin Zhao, Feiyan Zhao, Battogtokh Chimeddorj, Zhihong Sun, Enkhtsetseg Tserenkhuu, Munkhtsetseg Ochirdanzan, Dulamsuren Ganpurev, Weng Fun, Wusigale, Weicheng Li, Wenjun Liu, Shuying Yang, Mengdi Zhang, Enkhmaa Davaasambuu, Yagaantsetseg Talkhaa, Yansanjav Narankhuu, Sabri Bromage, Christina Warinner, Bilige Menghe, Davaasambuu Ganmaa

**Affiliations:** ^1^ Inner Mongolia Key Laboratory of Dairy Biotechnology and Engineering Hohhot Inner Mongolia China; ^2^ Key Laboratory of Dairy Products Processing, Ministry of Agriculture and Rural Affairs Hohhot Inner Mongolia China; ^3^ Key Laboratory of Dairy Biotechnology and Engineering, Ministry of Education Inner Mongolia Agricultural University Hohhot Inner Mongolia China; ^4^ Mongolian National University of Medical Sciences Ulaanbaatar Mongolia; ^5^ Mongolian Health Initiative Ulaanbaatar Mongolia; ^6^ Global Laboratory Ulaanbaatar Mongolia; ^7^ Maternal and Child Health Center Ulaanbaatar Mongolia; ^8^ Institute of Nutrition, Mahidol University Nakhon Pathom Thailand; ^9^ Department of Nutrition Harvard T. H. Chan School of Public Health Boston Massachusetts USA; ^10^ Department of Archaeogenetics Max Planck Institute for Evolutionary Anthropology Leipzig Germany; ^11^ Department of Anthropology Harvard University Cambridge Massachusetts USA; ^12^ Channing Division of Network Medicine, Brigham and Women's Hospital, Harvard Medical School and Harvard T.H. Chan School of Public Health Boston Massachusetts USA

**Keywords:** dietary nutrition, gut microbiota, health status, Mongolian population

## Abstract

Until recently, nomadic nomadism has been the dominant culture in Mongolia. Dietary patterns have evolved to fit this culture and ensure the health of people. This cross‐sectional study was conducted to investigate the key role of dietary nutrition in maintaining the health of the Mongolian population and its impact on gut microbiota. Meanwhile, the correlations between the gut microbiota, dietary nutrition, and health status of the Mongolian population were explored. This study revealed distinct patterns in the dietary structures of urban and nomadic populations. During winter, urban populations consume more fruits, vegetables, and egg products, whereas nomads consume more dairy products. The intake of nutrients such as energy, protein, and carbohydrates, and blood indices such as blood glucose and total cholesterol (TC) of nomadic populations were found to be significantly higher than those of urban residents (*p* < 0.05), and these nutrients exhibited significant correlations with the blood indices. Furthermore, the influence of both region and season on the gut microbiota of the Mongolian population with regional disparities was greater than that of seasonal variations. In winter, the gut microbiota composition of nomadic populations differed significantly from that of urban populations, as evidenced by a decrease in *Bacteroides*, *Phocaeicola*, *Phocaeicola vulgatus*, *Bacteroides uniformis* , and *Lachnospira eligens* and an increase in *Firmicutes*, *Alistipes*, *Dorea*, *Ruminococcus torques* , and *Dorea formicigenerans* (*p* < 0.05). Additionally, lactic acid bacteria and *Bifidobacterium* sp. were abundant in the gut of the Mongolian population, which present promising opportunities for developing and utilizing unique probiotic resources in Mongolia. The study also found correlations between microbial species and blood indices, as well as nutrients, providing novel evidence to support the complex relationships between gut microbiota, nutrition, and health status in the Mongolian population. Overall, this study revealed significant differences in dietary nutrition, gut microbiota, and health status among geographically diverse populations in Mongolia and contributed to a deeper understanding of the complex interplay between gut microbiota, nutrition, and health among Mongolians.

## Graphical abstract

Nomadic nomadism has been the dominant culture in Mongolia. Dietary patterns have evolved to fit this culture and ensure the health of Mongolia populations. The study revealed significant differences in dietary nutrition, gut microbiota, and health status among geographically diverse populations in Mongolia, as well as correlations between microbial species and blood indices. This contributes to a deeper understanding of the complex interplay between gut microbiota, nutrition, and health among Mongolians.

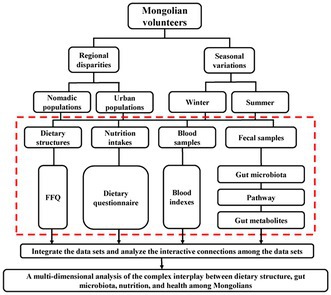

## 1 Introduction

Diet is a fundamental aspect of good health, and it is crucial to recognize its significance. In numerous regions worldwide, including Mongolia (Siracusa et al. 2023), rapid urbanization and the proliferation of processed foods have profoundly impacted dietary habits. A growing preference for foods high in calories, fat, sugar, and salt has emerged, and the baseline intake levels of fruits, vegetables, and dietary fiber are often insufficient (Dominguez et al. 2024; Popkin 2011). The community of microorganisms found in the human intestine is collectively referred to as the gut microbiota, which produces numerous metabolic products that play vital roles in the functioning of an organism (Luanfeng Wang et al. 2023). The gut microbiota produces various metabolic products that are essential for nutrient absorption (Danneskiold‐ Samsøe et al. 2019), intestinal barrier maintenance (Jiang et al. 2023), and regulation of intestinal immune function (Li et al. 2016).

The structure of the gut microbiome is significantly influenced by dietary composition (Zmora et al. 2019). For example, vegetarians exhibit a higher abundance of operational taxonomic units (OTUs) and greater microbial richness in the gut than omnivores (Veca et al. 2020). A high‐fiber diet promotes the growth of *Bifidobacterium* (Bervoets et al. 2013), and long‐term consumption of complex carbohydrates has been shown to promote the growth of *Prevotella* (Kolodziejczyk et al. 2019). Dietary patterns often vary depending on geographical factors. For instance, the Hadza people of Tanzania primarily consume raw and unprocessed foods, leading to a significantly higher diversity of gut microbiota than Westerners, whose diets are predominantly composed of commercial agricultural products (Smits et al. 2017). Moreover, geography has been identified as one of the most significant determinants of gut microbiota among rural and urban populations in South Africa (Tamburini et al. 2022). Seasonal effects also play a crucial role in this process. During summer, people tend to consume a lighter diet with more fruits and vegetables, leading to an abundance of *Actinobacteria*, which aids in the digestion of complex carbohydrates (Smits et al. 2017). Geography and season play significant roles in shaping the gut microbiota by influencing dietary changes. A diet that meets these basic requirements must include proteins, carbohydrates, minerals, vitamins, fibers, and water. The gut microbiota influences health by contributing to nutrient metabolism. For example, human milk contains a significant amount of “human milk oligosaccharides” (HMOs). These HMOs resist breakdown by gastric acid and digestive enzymes and contribute to the growth of beneficial bacteria such as Bifidobacterium in the gut of infants. Bifidobacteria ferment HMOs and produce “short‐chain fatty acids” (SCFAs), which help reduce intestinal pH, thereby inhibiting the growth of pathogenic bacteria. A deficiency in dietary fiber can significantly reduce the growth of Bacteroidetes and increase the levels of Proteobacteria in the gut (Thomson et al. 2018). This imbalance may contribute to cognitive impairment by altering the gut microbiota‐hippocampal axis (Shi et al. 2021). Although there is a growing understanding of the relationships between nutrient intake, gut microbiota, and health, but there remains very little is known about these relationships in Mongolia.

Mongolia is an expansive country characterized by diverse terrain and has successfully preserved its various natural features. It is one of the few countries where nomadic nomadism is still widely practiced, providing a livelihood for approximately 40% of the population (Jeong et al. 2020). Owing to factors such as geography and seasonal variations, the development of settled agricultural and animal husbandry sectors in Mongolia remains largely undeveloped. The Mongolian diet primarily consists of beef and mutton, which are consumed as staples year‐round, alongside dairy products and grains, which are considered non‐staple foods. However, imported fruits and vegetables also play a significant role in the Mongolian diet. Nomadic dietary practices are uncomplicated and largely centered on mutton cooked in clear water and naturally fermented dairy products such as yogurt, koumiss, milk curd, and hard cheeses. Urban residents, who have better access to transportation and grocery stores, enjoy a more diverse range of daily fruits, vegetables, and fast‐food items, such as instant noodles, hamburgers, and sandwiches. These urban–rural differences make Mongolia a suitable place for studying the interaction of location and season on gut microbiota (Zhang et al. 2014). Although a substantial body of evidence demonstrates that dietary patterns significantly influence gut microbiota, research on the specific characteristics of dietary patterns and gut microbiota in Mongolian residents remains limited. To address this gap, we conducted a study involving healthy Mongolian participants who resided in various regions of Mongolia and experienced different seasonal conditions throughout the year. Given the distinctive dietary patterns of Mongolian populations, we hypothesized that this traditional dietary structure significantly shapes the gut microbiota composition. We further posited that regional and seasonal fluctuations in dietary practices would lead to corresponding shifts in gut microbial profiles and that specific microbial signatures would mediate the observed associations between dietary structure and health outcomes. Our study aimed to evaluate the nutritional and health status of these populations, analyze the characteristics of their gut microbiota, and establish correlations among gut microbiota composition, nutrition, and health status in the Mongolian population. This study provides valuable insights that can serve as a robust reference for Mongolian individuals seeking to mitigate disease and improve health through dietary adjustments and interventions targeting the gut microbiota in the future.

## 2 Material and Methods

### 2.1 Ethics Approval and Consent to Participate

This study was conducted in accordance with the guidelines outlined in the Declaration of Helsinki and regulations of Good Clinical Practice (GCP). All procedures involving research study participants were approved by the Scientific Review Committees of the Mongolian National University of Medical Sciences and the Institutional Review Board of the Mongolian Ministry of Health, along with Harvard T.H. Chan School of Public Health Institutional Review Board (Protocol Title: “The study of the nutritional status of the Mongolian population and the biological diversity of intestinal lactobacillus”; Protocol Number: IRB20‐1749). All eligible participants provided written informed consent before participation in the study.

### 2.2 Experimental Design and Subject Recruitment

The regions selected for the study were the capital city of Mongolia, Ulaanbaatar, the southern province of Omnogobi, the north‐central province of Bulgan, the northern province of Khuvsgul, and the eastern province of Dornogobi. The Ulaanbaatar region was sampled during both summer and winter, whereas the rural regions were sampled only during winter. Participants were selected from the nomadic areas of Omnogobi, Bulgan, Khuvsgul, and Dornogobi. The remaining participants were urban residents of Ulaanbaatar, the capital of Mongolia. Given that dietary composition, particularly the consumption of milk and dairy products, is a central focus of this study and a historically significant element in both nomadic and urban Mongolian diets (Bromage et al. 2020), the sample size calculations were based on estimated dairy intake from prior research. Drawing upon comparative dietary data (Hess et al. 2021; Kakkoura et al. 2022), we assumed a mean intergroup dietary difference of 2.9 units, with a standard deviation of 5 units. To detect this difference at a two‐sided significance level of 5% (α = 0.05) and a statistical power of 90% (β = 0.2), a minimum of 77 participants per group was required. This calculation accounted for an anticipated dropout rate of less than 20%. Furthermore, we deliberately oversampled urban participants to ensure sufficient statistical power for subgroup analyses, acknowledging the higher variability in dietary patterns observed in the urban population.

Mongolian males and females residing in the above areas were screened using the inclusion and exclusion criteria. The inclusion criteria were 15–65 years old males or females residing in Mongolia (urban or nomadic area) for more than three years with no history of tourism or relocation during this period, agreed to participate in the study, and voluntarily signed an informed consent form. The exclusion criteria were a history of major chronic conditions, such as gastrointestinal and mental diseases or malignant tumors requiring hormonal drugs or immunosuppressants; receiving antibiotic treatment or supplements in the last three months; and declining participation in the study. A total of 302 healthy Mongolian individuals were selected as study participants (nomadic populatio*n* = 85, urban population = 217). The selection of the 302 participants was based on financial feasibility and understanding of the nutritional differences between the target populations of Ulaanbaatar as an urban region and Khangai and Gobi as rural regions. Table S1 on the participants.

### 2.3 Dietary Survey, Health Examination and Sample Collection

The study commenced with documentation of the participants' basic characteristics. Prior to administering the questionnaire, content validity was assessed by research teams situated in Ulaanbaatar and piloted among staff at the Mongolian Health Initiative. The Food Frequency Questionnaire (FFQ) was meticulously guided by research assistants who carefully recorded the consumption frequency of each food item. The reference frequencies varied from “never or < 1 per month”, “1‐3 per month”, “1 per week”, “past 2–4 per week”, “5‐6 per week”, “1 per day”, “2–3 per day”, and “4‐5 per day”, to “6+ per day”. Food categories were subdivided into 150 types, including Milk and Dairy products, eggs and egg products, condiments, beverages, alcoholic beverages, convenience food, stuffing‐type food, fruits and vegetables and nuts, chocolate products and sweets, cereal products, meat and meat products, and fish and aquatic products. The daily food consumption of the Mongolian population was determined using a dietary questionnaire. The Feihua Nutrition Calculator (Beijing Bowen Shixun Technology Co. Ltd., China) was used to convert daily food consumption into nutrient intake. A structured food nutrient composition database was constructed using authoritative sources, namely, the USDA FoodData Central and the Chinese Food Composition Table (Standard Edition). The nutrient and trace element contents of each food item were computed by cross‐referencing the intake quantities with this database. To ensure precision, adjustments for cooking losses and nutrient bioavailability were implemented using multi‐tiered correction procedures. In total, 370 FFQ and 370 dietary questionnaires (nomadic winter = 85, urban winter = 135, and urban summer = 150) were collected and recorded (Table S1).

A total of 185 venous blood samples (nomadic winter = 51, urban winter = 67, urban summer = 67) were collected from participants to evaluate their health status using blood biochemical indices. 5 mL of blood sample was collected and analyzed for various blood biochemical indexes including blood glucose (mmol/L), cholesterol (mg/dL), total cholesterol (TC, mg/dL), triglyceride (TG, mg/dL), high‐density lipoprotein cholesterol (HDL‐C, mg/dL), low‐density lipoprotein cholesterol (LDL‐C, mg/dL), nonhigh‐density lipoprotein cholesterol (non‐HLD‐C, mg/dL), HDL/LDL, glutamic pyruvic transaminase (ALT, U/L), glutamic oxaloacetic transaminase (AST, U/L), alkaline phosphatase (ALP, U/L), γ‐ glutamyl transferase (GGT, U/L), total protein (TP, g/L), albumin (ALB, g/L), bilirubin total Gen.3 (BILT3, mg/dL), bilirubin direct Gen.2 (BILD2, mg/dL), serum creatinine (Scr, mg/dL), urea (mg/dL), urine acid (UA, mg/dL), immunoglobulin G2 (IgG2, g/L), and C‐reaction protein 4 (CRP4, mg/L). Additionally, 185 fecal samples were collected from the participants using a Longseegen stool storage kit (Longsee, China). The samples were divided into the same three groups as the blood samples. The participants received brief training on the use of aseptic fecal samplers to prevent contamination during the sampling process. All fecal samples were marked, transported back to the laboratory, and stored at −80°C for further analysis (Table S1).

### 2.4 Extraction of DNA, Metagenomic Sequencing and Droplet Digital PCR

Metagenomic DNA from fecal samples was extracted using the QIAamp Fast DNA Stool Mini kit (Qiagen GmbH, Hilden, Germany) following the manufacturer's instructions. The quality of the extracted DNA was measured using a Nanodrop spectrophotometer and the integrity of the extracted DNA was evaluated using 1.0% agarose gel electrophoresis. The purity of the extracted DNA was then measured using the Qubit dsDNA Assay Kit with a Qubit 2.0 fuorometer (Life Technologies, CA, USA). DNA of high quality (DNA concentration greater than 20 ng/μL and OD260/280 between 1.8 and 2.0) was selected and used with the NEBNext Ultra DNA Library Prep Kit for Illumina (New England Biolabs Inc., USA) to construct sequencing libraries. Sequencing libraries were constructed according to the manufacturer's instructions. Finally, sequencing libraries were sequenced on an Illumina NovaSeq platform to generate paired‐end reads (Tianjin Novogene Technology Co. Ltd., Tianjin, China).

Droplet digital PCR was used to quantitatively analyze lactic acid bacteria in the gastrointestinal tract of the Mongolian population. This method eliminates the need for standard curves and control samples and treats each reaction as an independent system. Calculations were performed to obtain the copy numbers of the samples and ensure accurate results for absolute quantitative analysis. Therefore, dd‐PCR is widely used in microbial testing. According to Hou's study (Hou et al. 2018), the specific quantitative primers for lactic acid bacteria were determined (WLAB1: TCCGGATTTATTGGGCGTAAAGCGA; WLAB2: TCGAATTAAACCACATGCTCCA) and used in conjunction with dd‐PCR. The components of the dd‐PCR system were as follows: WLAB1, 0.2Μl; WLAB2, 0.2 μL; DNA, 2 μL; ddH_2_O, 7.6 μL; QX200 dd‐PCR EvaGreen SuperMix, 10 μL; and Droplet Generation Oil for EvaGreen, 70Μl. The dd‐PCR program consisted of the following steps: 95°C for 10 min; 94°C for 30 s and 55°C for 1 min, repeated for a total of 40 cycles; 4°C for 5 min; 90°C for 5 min; and 12°C for maintenance. After the PCR procedure, a QX 200TM Droplet Reader was used to read the amplified droplets, and the results were calculated according to formula 1.

Formula 1:

$$N\left(\frac{\mathrm{CFU}}{g}\right)=\frac{20*\text{solution volume}\left(\mathrm{\mu L}\right)*\text{dilution factor}*\text{copy number}\;\left(\;\text{copies}/\mathrm{\mu L}\;\right)}{2*\text{sample mass required for}\;\mathrm{DNA}\;\text{extraction}\;\left(g\right)}$$

### 2.5 Quality Control of Metagenome Data

A total of 185 fecal samples were subjected to shotgun sequencing, resulting in the generation of 1.218 Tb of paired‐end reads (6.586 ± 0.312 Gb per fecal sample; range = 5.868–7.450 Gb). To remove low‐quality sequences and host‐contaminated reads, Bowtie2 (v.2.4.4) and Trimmomatic were used with the KneadData quality control pipeline (http://huttenhower.sph.harvard.edu/kneaddata). After processing, 1.200 Tb of high‐quality paired‐end reads (6.486 ± 0.310 Gb per fecal sample; range = 5.770–7.378 Gb) remained for downstream analysis (Table S3).

### 2.6 Bioinformatics of Metagenomic Data

Taxonomy, functional genes, and metabolic pathway annotation of high‐quality paired‐end reads were performed using HUMAnN3.0 (https://huttenhower.sph.harvard.edu/humann/). MetaPhAn3 was used to determine species abundance. Gene families and metabolic pathways were analyzed as follows: bowtie2 (v.2.4.4) was used to compare pan‐genomic nucleotide sequences of the samples using the ChocoPhIAn3 tool. Diamond was used to translate unaligned reads into protein sequences. Subsequently, protein sequences were compared using UniRef90. Genome and protein sequences were calculated using the HUMAnN core algorithm and MetaCyc database. Finally, the abundance of gene families and coverage of metabolic pathways were analyzed.

### 2.7 Prediction of Gut Active Metabolites

MelonnPan was used to predict the active gut metabolites. To obtain high‐quality sequences, the seqtk program (https://github.com/lh3/seqtk) and the blastx function of DIAMOND with parameters –query‐cover 90 and id 50 were used to extract 1 million reads from each sample. These sequences were screened to obtain hits for the respective genes, and gene abundance in each sample was calculated using the best hit for each gene. Gene abundance was subsequently converted into predicted active metabolite profiles using the MelonnPan‐predicted pipeline.

### 2.8 Statistical Analysis

All statistical analyses were performed using R software (v.4.2.1) and STAMP software (v.2.1.3, Australian Center for Ecogenomics, Australia), and the data were expressed as average averages. The Shannon and Chao 1 indices were calculated using R packages (including vegan, ggpubr, and dplyr) to evaluate microbial diversity in fecal samples. Principal coordinate analysis (PcoA, based on Bray–Curtis distance) was used to evaluate the microbial structure in fecal samples, and analysis of similarities (PERMANOVA; 999 permutations) was performed to evaluate differences between the two groups. Significant differences between groups were calculated using the Wilcoxon test (cutoff level, *p* < 0.05). Procrustes analysis was used to assess consistency between the two datasets. Correlations between key species in the gut microbiota and indices were identified based on a mental test. All graphical presentations were generated using R and Adobe Illustrator (AI).

## 3 Results

### 3.1 The Frequency of Urban Population Eating Fruits, Vegetables and Egg Products Was Significantly Higher Than That of Nomadic Population in Winter and Urban Population Prefer Convenience Food in Summer

The FFQ was used to investigate the characteristics of the dietary structure and compare the differences between the diets of Mongolian nomadic and urban populations. The monthly frequency of consumption of eggs and egg products, convenience food, fruits and vegetables, nuts, chocolate products, sweets, fish, and aquatic products was significantly higher in the winter urban population than that in the winter nomadic population (*p* < 0.05). However, during the winter months, the nomadic population consumed more milk and dairy products, particularly milk tea, fresh milk, and clotted milk products, as well as more alcoholic beverages, such as koumiss, than the urban population. In general, the urban population consumed fruits, vegetables, eggs, candy, and chocolate products more frequently than the Mongolian nomadic population. Furthermore, during the winter months, the urban population consumed fewer convenience foods and more cereals, meat, and meat products than during the summer months (*p* < 0.05). The consumption of other food items showed only slight variations (Table 1; Table S2).

**TABLE 1:** FFQ of healthy Mongolian population.

Food frequency questionnaire	Nomadic winter	Urban winter	Urban summer	*p*	*p*
(per month)	(*n* = 85)	(*n* = 135)	(*n* = 150)	NW vs UW	UW vs US
Milk and Dairy products	8.321	3.914	3.333	1	1
Eggs and egg products	3.514	5.896	5.570	< 0.001	0.23
Condiments	1.391	2.136	2.523	0.1	0.49
Beverage	6.835	7.497	8.685	0.79	0.47
Alcoholic beverage	3.057	2.711	2.942	0.11	0.39
Convenience food	0.489	1.457	1.930	< 0.001	0.002
Stuffing type food	2.615	3.017	2.805	0.07	0.22
Fruits, vegetables and nuts	1.961	2.669	2.363	< 0.001	0.08
Chocolate products and sweets	2.488	3.422	3.724	< 0.001	0.47
Cereal product	4.912	4.168	3.554	0.05	< 0.001
Meat and meat products	2.218	2.247	1.923	0.82	0.02
Fish and aquatic products	1.011	2.126	1.936	0.003	0.18

*Note:* Expressed as the average of each group. The significant difference intergroup were calculated by Wilcoxon tests (cutoff level: *p* < 0.05).

Abbreviations: FFQ, Food frequency questionnaire; NW, nomadic winter; US, urban summer; UW, urban winter.

### 3.2 Intake of Energy, Protein, Carbohydrate and Dietary Fiber in Nomadic Population Were Significantly Higher Than That in Urban Population

The findings from the analysis of the daily nutrient intake of the healthy population in Mongolia indicate that, although there were some individual variations among certain individuals, the energy, protein, carbohydrate, dietary fiber, vitamin A, vitamin B1, vitamin B2, calcium, phosphorus, potassium, magnesium, iron, zinc, selenium, copper, and manganese intakes of the nomadic population in winter were significantly higher than those of the urban population (*p* < 0.05). Conversely, the vitamin C and iodine intakes of the urban population in winter were significantly higher than those of the nomadic population (*p* < 0.05). Additionally, the intake of protein, dietary fiber, vitamin D, vitamin E, niacin, calcium, phosphorus, potassium, magnesium, iron, zinc, selenium, copper, and manganese in the urban population in winter was significantly higher than that in summer (*p* < 0.05; Table 2). These results indicate that dietary differences play a significant role in the disparities in nutrient intake between urban and nomadic Mongolian populations.

**TABLE 2:** Mean usual intake of nutrients in one day of healthy Mongolian population.

Mean usual intake of nutrients	Nomadic winter	Urban winter	Urban summer	*p*	*p*
(per day)	(*n* = 51)	(*n* = 67)	(*n* = 67)	NW vs UW	UW vs US
Energy (Kcal)	3002.392	2286.333	1878.935	0.009	0.15
Protein (g)	199.453	124.492	84.693	< 0.001	0.01
Fat (g)	54.276	51.124	41.730	0.22	0.819
Carbohydrates (g)	445.986	338.424	286.715	0.015	0.145
Dietary Fiber (g)	124.461	81.038	33.184	0.009	0.003
Cholesterol (mg)	283.784	329.892	308.302	0.66	0.56
Vitamin A (μgRAE)	2045.255	1106.348	1327.283	< 0.001	0.982
Vitamin D (μg)	3.565	3.139	2.057	0.08	0.024
Vitamin E (μg)	24.703	19.673	9.798	0.12	0.017
Vitamin B1 (mg)	1.356	0.977	1.096	0.02	0.207
Vitamin B2 (mg)	2.175	1.201	0.877	< 0.001	0.093
Vitamin B6 (mg)	0.183	0.238	0.288	0.66	0.522
Vitamin C (mg)	41.429	58.939	29.562	0.036	0.236
Folic acid (μg)	61.794	65.885	85.359	0.28	0.25
Niacin (mg)	39.116	42.600	20.813	0.085	0.009
Choline (mg)	67.931	122.619	86.125	0.99	0.335
Biotin (μg)	55.417	51.106	25.226	0.93	0.16
Calcium (mg)	2020.098	917.773	476.891	< 0.001	0.018
Phosphorus (mg)	2525.392	1540.939	979.500	< 0.001	0.009
Potassium (mg)	8833.569	5501.258	2220.022	< 0.001	0.001
Sodium (mg)	557.890	593.535	623.930	0.61	0.343
Magnesium (mg)	1152.378	635.894	343.196	< 0.001	0.002
Iron (mg)	119.344	55.371	20.200	< 0.001	0.005
Iodine (mg)	6.786	39.728	21.088	< 0.001	0.21
Zinc (mg)	38.139	18.425	11.142	< 0.001	0.007
Selenium (mg)	197.544	118.665	56.375	0.011	0.017
Copper (mg)	12.204	6.400	2.984	< 0.001	0.005
Manganese (mg)	228.172	84.964	26.232	< 0.001	0.006

*Note:* Expressed as the average of each group. The significant difference intergroup were calculated by Wilcoxon tests (cutoff level: *p* < 0.05).

Abbreviations: NW, nomadic winter; US, urban summer; UW, urban winter.

### 3.3 Significant Differences in Health Status Between the Nomadic and Urban Populations, and the Nutrient Intake and Blood Indexes Showed High Consistency

**FIGURE 1:**
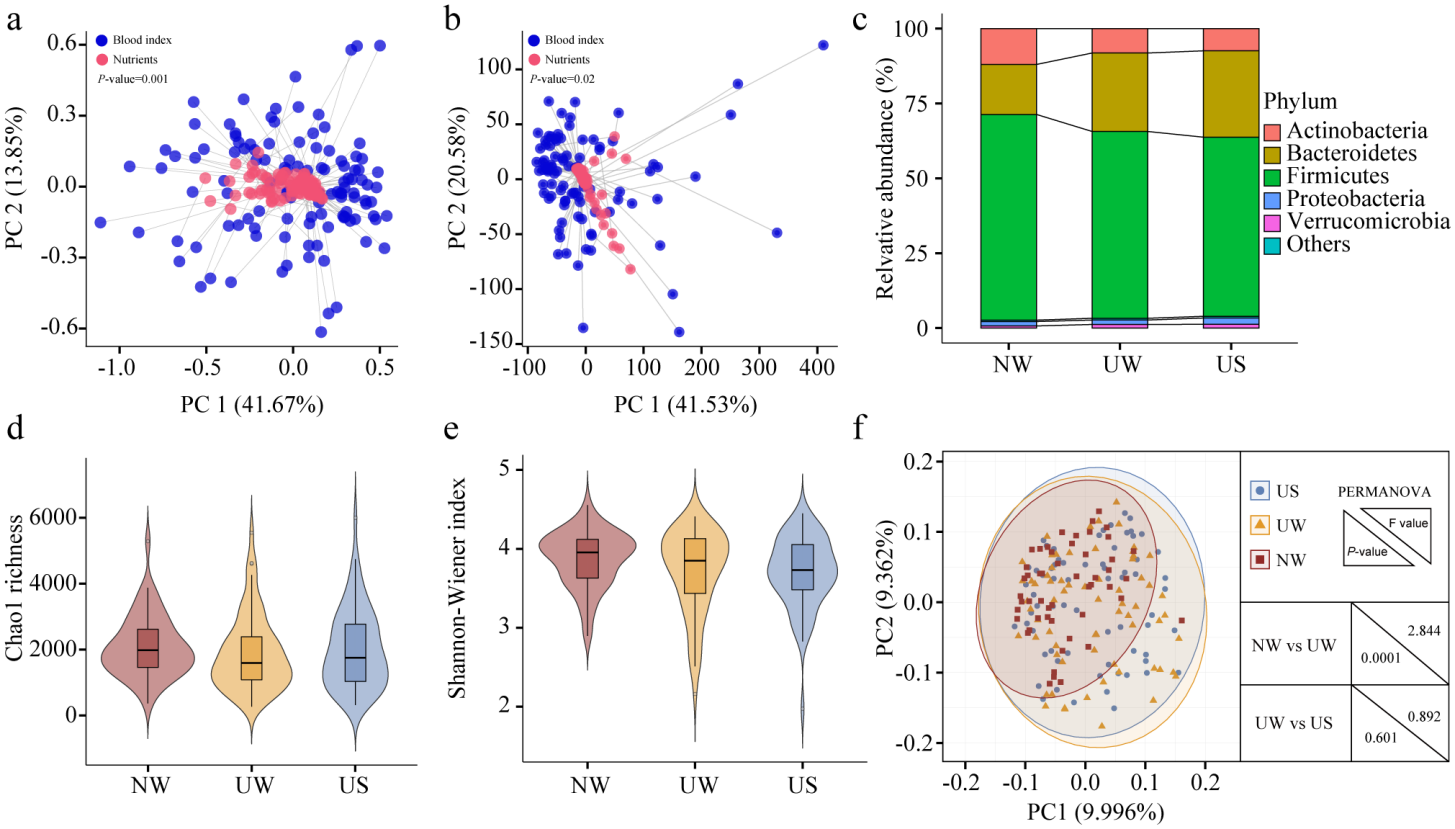
(a‐b) Consistency between blood indices and nutrient intake in the Mongolian population. Blue and red represent the blood indices and nutrient intake, respectively. (c) Composition of dominant phyla in the different groups. Each phylum is represented by a different color. (d‐f) Microbial diversity in the Mongolian population. Chao1 richness and Shannon–Wiener index of fecal microbiome in NW, UW, and US, respectively. The violin plot represents the distribution state of each group of data, and the horizontal line inside the box represents the median. Principal coordinate analysis (PCoA) scores for the three groups. Red, yellow, and blue represent NW, UW, and US, respectively. NW, UW, and US represent nomadic winter, urban winter, and urban summer, respectively.

The health status of the participants was assessed by monitoring their blood glucose, blood lipids, liver function, kidney function, and immune indices. In winter, the nomadic population had higher blood glucose, total cholesterol (TC), low‐density lipoprotein cholesterol (LDL‐C), non‐high‐density lipoprotein cholesterol (non‐HDL‐C), HDL/LDL ratio, alkaline phosphatase (ALP), urea, and IgG2 levels than the urban population (*p* < 0.05). Furthermore, HDL‐C levels in the urban population were significantly higher than those in the nomadic population (*p* < 0.05). These findings indicate significant differences in health status between nomadic and urban Mongolian populations. Specifically, blood glucose, HDL‐C, LDL‐C, ALT, and BILT3 levels in the urban population were significantly higher in winter than in summer (*p* < 0.05). In contrast, TC, ALP, GGT, TP, ALB, Scr, UA, and IgG2 levels in summer were significantly higher than those in winter (*p* < 0.05, Table 3), and procrustes analysis demonstrated a significant correlation between nutrient intake and blood indices (*p* < 0.05, Figure 1a,b). These results confirm that nutrient intake is closely related to health and that maintaining a balanced nutrient intake is crucial for the well‐being of the Mongolian population.

**TABLE 3:** Blood index of healthy Mongolian population.

Blood index	Nomadic winter	Urban winter	Urban summer	*p*	*p*
	(*n* = 51)	(*n* = 67)	(*n* = 67)	NW vs UW	UW vs US
Blood glucose (mmol/L)	6.090	5.454	5.209	0.02	0.034
Cholesterine (mg/dL)	170.292	166.119	164.448	0.29	0.643
TC (mg/dL)	122.042	101.701	125.418	0.004	0.003
TG (mg/dL)	26.046	24.876	26.226	0.22	0.38
HDL‐C (mg/dL)	46.625	59.194	53.863	< 0.001	0.001
LDL‐C (mg/dL)	107.565	91.155	83.369	0.004	0.042
Non‐HLD‐C (mg/dL)	123.667	107.552	109.254	0.009	0.64
HDL/LDL	2.617	1.694	1.788	< 0.001	0.16
ALT (U/L)	19.659	16.994	13.548	0.84	0.015
AST (U/L)	24.600	20.316	20.752	0.72	0.733
ALP (U/L)	82.137	60.791	67.672	< 0.001	0.001
GGT (U/L)	44.565	30.460	35.430	0.12	0.021
TP (g/L)	68.557	66.907	70.339	0.11	0.001
ALB (g/L)	40.447	41.109	43.425	0.64	< 0.001
BILT3 (mg/dL)	0.404	0.442	0.248	0.27	< 0.001
BILD2 (mg/dL)	0.157	0.148	0.118	0.31	0.11
Scr (mg/dL)	0.910	0.858	0.896	0.16	0.038
Urea (mg/dL)	27.890	23.576	24.990	0.003	0.177
UA (mg/dL)	5.182	5.070	5.463	0.45	0.005
IgG2 (g/L)	14.353	12.572	13.385	0.005	0.003
CRP4 (mg/L)	4.131	2.322	3.304	0.067	0.2

*Note:* Expressed as the average of each group. The significant difference intergroup were calculated by Wilcoxon tests (cutoff level: *p* < 0.05).

Abbreviations: NW, nomadic winter; US, urban summer; UW, urban winter.

### 3.4 Significant Differences in Gut Microbiota Structure and Composition Between Mongolian Nomadic Population and Urban Population

In winter, there were no significant intergroup differences in alpha diversity between the Mongolian population groups, as indicated by both Chao1 richness and the Shannon–Wiener diversity index. However, the diversity and richness of the gut microbiota in the nomadic population were marginally higher than those in the urban population (Figure 1d,e). Beta diversity results revealed a significant difference in the structure of the gut microbiota between the nomadic and urban populations, as determined by permutational multivariate analysis of variance (PERMANOVA, *p* = 0.0001; Figure 1f). These results showed that while regional differences did not have a significant impact on the diversity of the gut microbiota among healthy Mongolian populations, the gut microbiota of the nomadic population was more diverse and abundant. Furthermore, it is important to note that the overall structure of gut microbiota varies greatly among healthy Mongolian populations residing in different regions.

**FIGURE 2:**
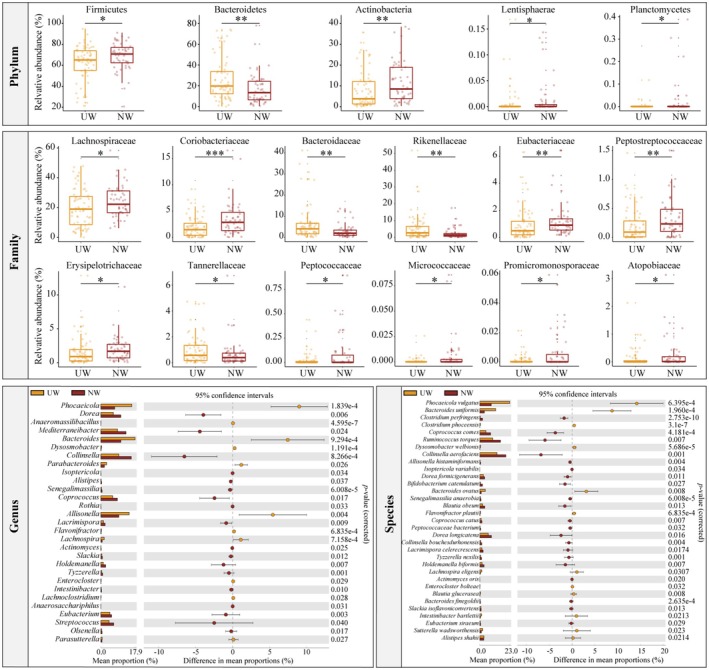
Significant differences in relative abundance were observed at the phylum, family, genus, and species levels between the urban and nomadic populations in winter. At the phylum and family levels, the horizontal line inside the box represents the median, and each dot represents a participant's data point. Significant differences between groups are represented by * (*p* < 0.05) and ** (*p* < 0.01). At the genus and species levels, the columns in the bar chart represent the average values, and the values on the right represent significance. Red and yellow colors represent NW and UW, respectively. NW and UW represent nomadic and urban winter seasons, respectively.

To further explore intricate changes in the gut microbiota at a finer level, the relative abundance of bacteria was calculated for each taxonomic level between the groups. At the phylum level, the dominant phyla in the healthy Mongolian population were Firmicutes, Bacteroidetes, Actinobacteria, Proteobacteria, and Verrucomicrobia, with relative abundances of greater than 1% (Figure 1c). In the urban population during winter, the relative abundance of Bacteroides in the gut was significantly higher than that in the nomadic population (*p* < 0.05), whereas the relative abundance of Firmicutes, Actinobacteria, Lentisphaerae, and Planctomycetes in the gut of the nomadic population was significantly higher than that in the urban population (*p* < 0.05). At the family level, 53 families had an occurrence rate higher than 30% in the gut, among which 14 families exhibited significant differences between the nomadic and urban populations. The relative abundances of Bacteroidaceae, Rikenellaceae, and Tannerellaceae in the gut of the urban population were significantly higher than those in the nomadic population (*p* < 0.05). Conversely, the relative abundance of Lachnospiraceae, Coriobacteriaceae, Eubacteriaceae, Peptostreptococcaceae, Erysipelotrichaceae, Peptococcaceae, Micrococcaceae, Promicromonosporaceae and Atopobiaceae in the gut of the nomadic population was significantly higher than that of the urban population (*p* < 0.05). In terms of genera, there were 155 genera with occurrence rates greater than 30% in gut, among which 29 genera showed significant differences between the nomadic population and urban populations. Notably, the relative abundances of *Bacteroides*, *Allisonella*, and *Lachnospira* in the gut of the urban population were significantly higher than those of the nomadic population (*p* < 0.05), whereas the relative abundance of *Coprococcus*, *Rothia*, and *Streptococcus* in the gut of the nomadic population was significantly higher than that of the urban population (*p* < 0.05). In terms of species, 265 species exhibited gut occurrence rates greater than 30%, of which 33 species showed significant differences between the nomadic and urban populations. Beneficial bacteria such as *Bacteroides uniformis* , *Bacteroides ovatus*, and *Blautia mucicerasia* were significantly enriched in the urban population (*p* < 0.05), whereas beneficial bacteria and short‐chain fatty acid (SCFAs)‐producing bacteria such as *Bacteroides ovatus* , *Bifidobacterium catenulatum* , *Coprococcus comes* , *Coprococcus catus* and *Holdemanella biformis* were significantly enriched in the nomadic population (*p* < 0.05). These results indicate that beneficial bacteria are present in the gut of healthy Mongolian populations across different regions, but regional differences contribute to significant variations in the composition of beneficial bacteria within the gut of healthy Mongolian populations (Figure 2).

In addition, there was no significant difference in the alpha and beta diversity of the gut microbiota in the urban population between winter and summer (Figure 1d–f). At the family level, the relative abundances of Micrococcaceae and Promicromonosporaceae were significantly higher in summer than in winter (*p* < 0.05). The relative abundances of *E. coli, Lachnospira*, *Rothia*, and *Isoptericola* in summer were significantly higher than those in winter (*p* < 0.05), whereas the relative abundance of *Phocea* in winter was significantly higher than that in summer (*p* < 0.05). At the species level, the relative abundances of *Escherichia coli* , *Ruminococcus callidus* , and *Streptococcus oralis* in summer were significantly higher than those in winter (*p* < 0.05), whereas the relative abundances of *Phocea massiliensis* and *Isoptericola variabilis* in winter were significantly higher than those in summer (*p* < 0.05; Figure S1). In summary, both region and season could affect the composition of the gut microbiota of the Mongolian population; however, the influence of geographical region on the gut microbiota was greater than that of season.

### 3.5 The Gut Microbiota of the Mongolian Population Was Found to Be Rich in Lactic Acid Bacteria and Bifidobacterium Species

**FIGURE 3:**
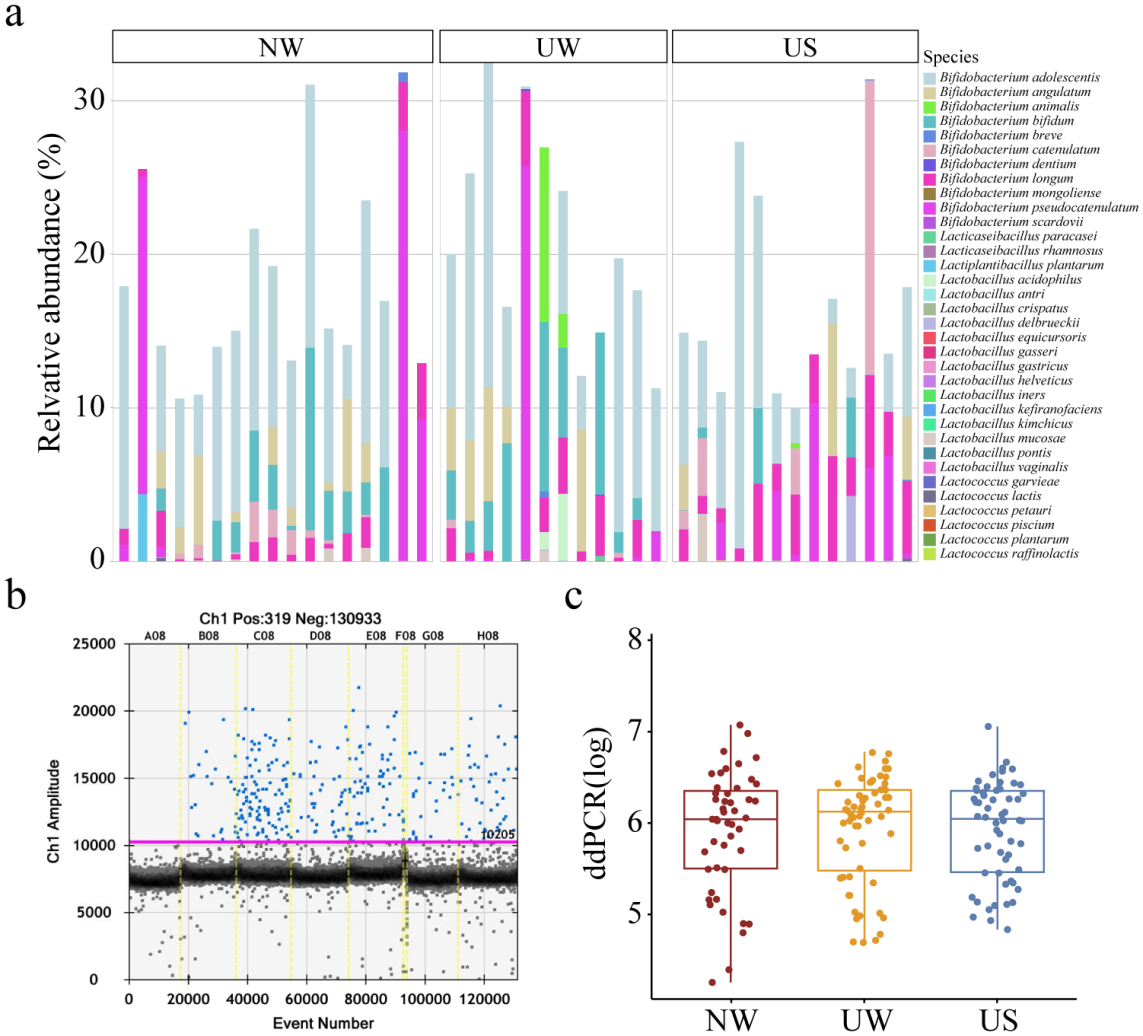
(a) Composition of lactic acid bacteria and *Bifidobacterium* sp. The stacking plot represents the cumulative abundance of lactic acid bacteria and *Bifidobacterium* sp. (b) Absolute quantification of lactic acid bacteria (LOG value calculated by dd‐PCR). Number of droplets Distribution of lactic acid bacteria in the gut of the Mongolian population. (c) Box plot showing the number of lactic acid bacteria in the gut of the Mongolian population. Red, yellow and blue represent NW, UW and US, respectively. NW, UW, and US represent nomadic winter, urban winter, and urban summer, respectively.

Our investigation revealed a substantial presence of lactic acid bacteria and *Bifidobacterium* spp. in the gut microbiota of healthy Mongolian individuals. A total of 34 species belonging to the genera *Lactobacillus*, *Lactococcus*, *Bifidobacterium*, *Lactiplantibacillus*, and *Lacticaseibacillus* were identified. Among the 185 fecal samples collected, the cumulative abundance of lactic acid bacteria and *Bifidobacterium* sp. was 10% in 42 samples (Figure 3a). Moreover, dd‐PCR results indicated no significant differences in lactic acid bacteria counts between the distinct regions and seasons in the Mongolian population (Figure 3b,c; Table S4).

### 3.6 Significant Differences Were Observed in Gut Metabolic Pathway Between Mongolian Nomadic Population and Urban Population

**FIGURE 4:**
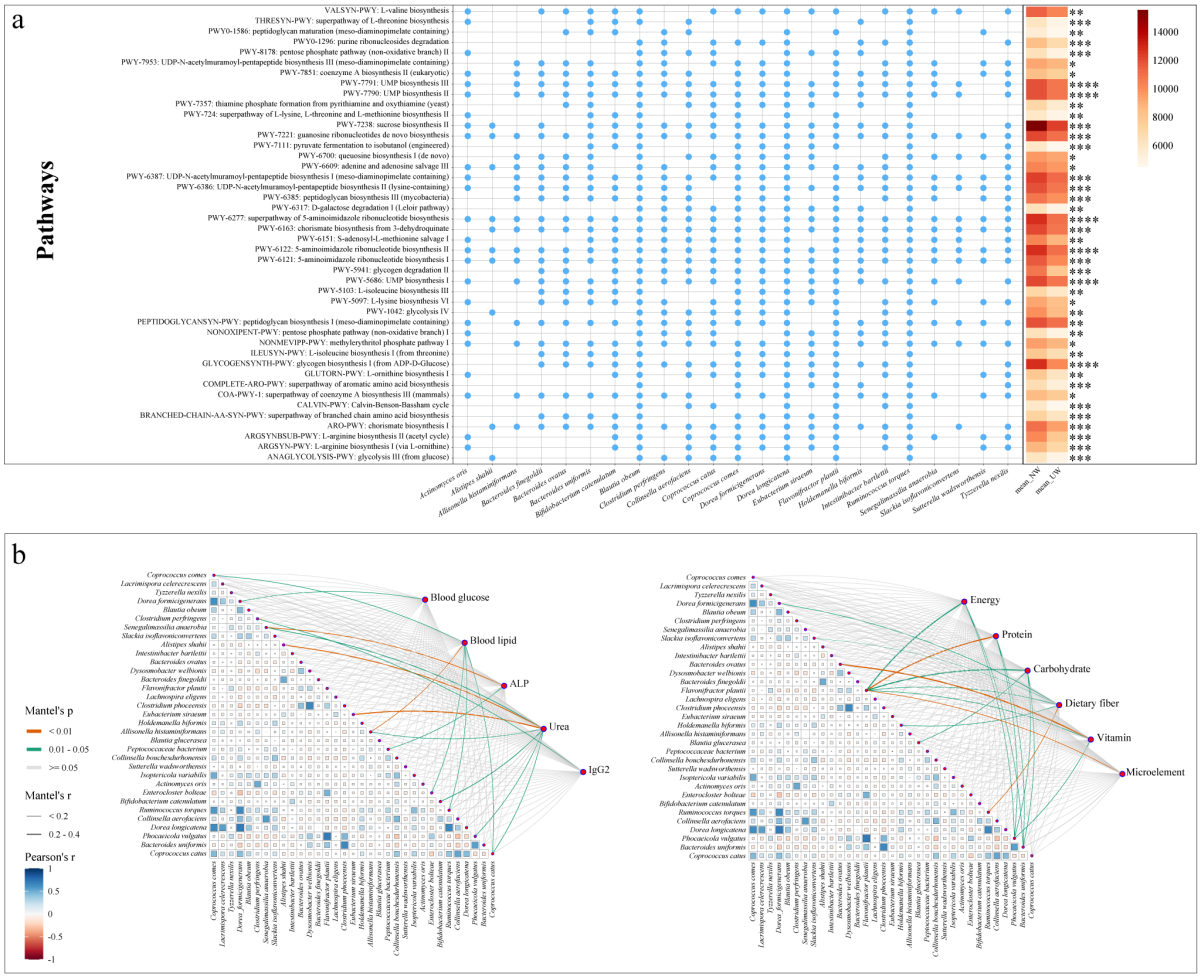
(a) Significantly different metabolic pathways between urban and nomadic populations in winter. Each dot represents the contribution of a species to the metabolic pathway. The colored blocks in the heat map represent the abundance of metabolic pathways. Significant differences between different groups are represented by * (*p* < 0.05), ** (*p* < 0.01), and *** (*p* < 0.001). (b) Correlation network plot of blood indices, nutrient intake, and differential microbiota species. The squares represent species correlations, and the lines represent correlations of differential species with blood indices and nutrient intake.

These findings indicate that region is a primary factor influencing the gut microbiota composition of a healthy population in Mongolia. Subsequently, we conducted a focused search for significant differential metabolic pathways between nomadic and urban Mongolian populations. Among the 516 monitored metabolic pathways, 60 exhibited an abundance exceeding 5000. Of these, 44 metabolic pathways were significantly different between the nomadic and urban populations in winter. Additionally, 23 significantly different species were found to be involved in the regulation of the metabolic pathways. The significantly different metabolic pathways mainly involved pyrimidine nucleotide synthesis (PWY‐5686, PWY‐7791, PWY‐7790), purine nucleotide synthesis and metabolism (PWY‐6121, PWY‐6122, PWY‐6277, PWY‐6609), energy synthesis and decomposition (GLYCOGENSYNTH‐PWY, ANAGLYCOLYSIS‐PWY, PWY‐1042, NONOXIPENT‐PWY, COA‐PWY‐1, PWY‐7111, PWY‐8178, PWY‐5941), peptidoglycan biosynthesis process (PEPTIDOGLYCANSYN‐PWY, PWY0‐1586, PWY‐6385, PWY‐6386, PWY‐6387, PWY‐7953), and amino acid synthesis and metabolism processes (COMPLETE‐ARO‐PWY, THRESYN‐PWY, ARGSYN‐PWY, ARGSYNBSUB‐PWY, PWY‐5103, PWY‐6151, PWY‐724). The abundance of all differential metabolic pathways was significantly higher in the nomadic population (*p* < 0.05, Figure 4a, Table S5). These results suggest that intestinal function is more active in nomadic populations.

### 3.7 Correlations Between Gut Microbiota, Nutrition and Health Status

To investigate the interactions between gut microbiota, nutrition, and health status, we examined the correlations between significantly different species and blood indices and daily nutrient intake. Our findings revealed that *Dorea formicigenerans* was positively correlated with blood glucose levels (*p* < 0.05). In addition, *Coprococcus comes* , *Coprococcus catus* , *Ruminococcus torques* , *Enterocloster bolteae*, and *Allisonella histaminiformans* were positively correlated with blood lipid levels (*p* < 0.05). *Peptococcaceae* and *Senegalimassilia anaerobia* were positively correlated with ALP (*p* < 0.05). Urea was positively correlated with *Bifidobacterium catenulatum* and *Alistipes shahii* (*p* < 0.05). Furthermore, *Clostridium perfringens* was positively correlated with IgG2 levels (*p* < 0.05, Figure 4a). Energy, protein, and carbohydrate intakes were positively correlated with *Flavonifractor plautii* , *Bacteroides uniformis*, and *Phocaeicola vulgatus* (*p* < 0.05). Dietary fiber intake was positively correlated with *Dorea formicigenerans* , *Ruminococcus torques* , *Holdemanella biformis*, *Flavonifractor plautii*, and *Slackia isoflavoniconvertens* (*p* < 0.05). Vitamin intake was positively correlated with *Bacteroides ovatus* , *Flavonifractor plautii*, and *Phocaeicola vulgatus* (*p* < 0.05). Trace element intake was positively correlated with *Flavonifractor plautii* abundance (*p* < 0.05, Figure 4b).

### 3.8 Significant Differences Were Observed in the Gut Bioactive Metabolome Between Mongolian Nomadic Population and Urban Population

**FIGURE 5:**
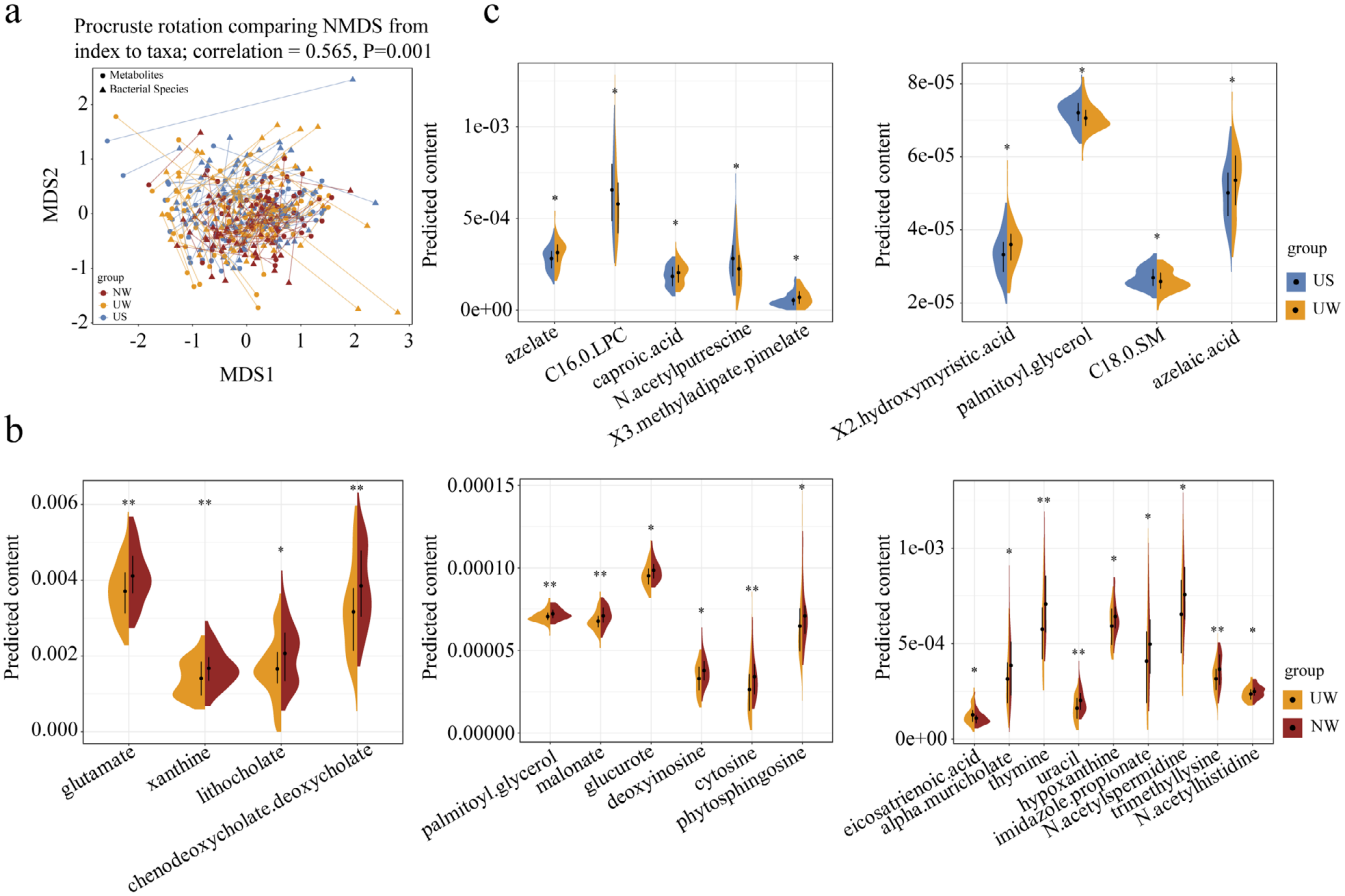
Prediction accuracy and differences in active intestinal metabolite levels (a) Procrustes analysis indicates the prediction accuracy of active intestinal metabolites. The connecting line represents the association between metabolites and species. (b, c) Significant differences in intestinal active metabolites among the three groups. The violin plot represents the distribution state of each group of data, and the horizontal dot represents the median. Significant differences between different groups are represented by * (*p* < 0.05) and ** (*p* < 0.01). Red, yellow and blue represent NW, UW, and US, respectively. NW, UW, and US represent nomadic winter, urban winter, and urban summer, respectively.

MelonnPan was used to predict the bioactive compounds in the gut, resulting in the identification of 80 metabolites. Procrustes analysis confirmed a good correlation between the gut microbiota and predicted gut metabolites (correlation = 0.565, *p* = 0.001). A total of 19 of the most abundant metabolites, including glutamate, xanthine, lithocholate, and chenodeoxycholate, differed significantly between the nomadic and urban populations in winter. The levels of glutamate, xanthine, lithocholate, chenodeoxycholate, deoxycholate, cytosine, uracil, thymine, deoxyinosine, and hypoxanthine in the gut of the nomadic population were significantly higher than those in the urban population (*p* < 0.05; Figure 5). These results indicate significant differences in the predicted gut metabolites among Mongolian populations residing in different regions. Interestingly, even when both populations were healthy, the nomadic environment significantly improved the gut metabolites of the population, which may be one of the factors contributing to the different health statuses of the urban and nomadic populations.

## 4 Discussion

There is a growing global recognition of the interconnections between general health, diet, and the gut microbiome. Gut microbiota have been implicated in various diseases, such as obesity (Delaye et al. 2023), diabetes (Han et al. 2023), heart disease (Karmazyn and Gan 2023), hypertension (O'Donnell et al. 2023), and gout (Zhao et al. 2023). Therefore, it is essential to improve our understanding of health by examining the effects of diet and other lifestyle factors on gut microbiomes. Our study demonstrated that Mongolian populations with different diets and health conditions have significantly distinct gut microbiota. The correlation between diet and health in the Mongolian population may be modulated by the gut microbiota. Among the Mongol groups, lifestyle differences (nomadic vs. urban) had a more significant influence on the gut microbiome than seasonal variations (winter vs. summer). Furthermore, we found a considerable presence of lactic acid bacteria and *Bifidobacterium* species in the gut of the Mongolian population, which reflects their dietary habits. This presents favorable conditions for the development and utilization of unique probiotic resources in Mongolia.

The Mongolian population has developed a distinctive dietary pattern owing to its unique geographical environment and lifestyle. Over time, nomadic populations in Mongolia have relied predominantly on meat and milk products, including staples such as milk tea, yogurt, and milk‐based beverages. However, fruits, vegetables, and grains are relatively scarce and are primarily imported, making them more accessible to urban residents. Our research confirms the persistence of these dietary patterns. Urban diets are more diverse and exhibit less seasonal variation than nomadic ones. City dwellers have greater access to convenience foods and specialty items, such as chocolate. Notably, the convenience of urbanized life partially diminishes the seasonal influences on diet. For the urban population, there was little difference in the dietary structure between winter and summer. However, our findings suggest that nomadic populations have a higher caloric intake of both proteins and carbohydrates, possibly because of the increased energy expenditure associated with their nomadic lifestyles.

Diet is known to play a predominant role in shaping the gut microbiome (Huang et al. 2022; Kolodziejczyk et al. 2019). For example, a high‐carbohydrate diet has been associated with higher levels of *Enterococci* (Kang et al. 2022). The Mediterranean diet has been reported to improve the balance of gut microbiota (Boughanem et al. 2023). Therefore, the observed regional and seasonal variability in the gut microbiota of healthy Mongolian individuals was not unexpected. Our findings suggest that the pronounced differences in dietary structure between urban and nomadic populations in Mongolia are key determinants of the distinct microbial profiles observed in these groups. Specifically, these dietary discrepancies contribute to substantial divergence in the structure and composition of gut microbiota. Notably, our data indicate that seasonal fluctuations in diet do not significantly affect the overall stability of gut microbiota in the Mongolian population. This resilience may be attributed to the traditional Mongolian diet, which predominantly consists of meat and dairy products. This diet, characterized by high and consistent protein and fat levels, provides a relatively stable nutritional milieu that supports a robust microbial ecosystem. Even in the face of seasonal changes that introduce modest variations in food types and nutrient intake, the gut microbiota exhibits remarkable adaptability. This adaptability is likely mediated by intrinsic microbial metabolic regulatory mechanisms that help preserve microbial homeostasis. Moreover, long‐standing environmental conditions, lifestyle practices, and genetic predispositions of Mongolian individuals further contribute to the enduring stability of the gut microbiome. These interconnected factors collectively enhance the resistance of the microbiota to seasonal dietary perturbations, thereby maintaining a relatively balanced intestinal ecosystem. While the gut microbiome of Mongols primarily comprises Firmicutes (F), Bacteroidetes (B), and Actinomycetes (A), the F/B ratio is higher in the nomads. This ratio is often used to assess gut microbiota and is mainly influenced by the dietary habits and health status of the host (Yañez et al. 2021). Nagawa et al. found that the F/B ratio in obese adults and children was higher than that in normal‐weight individuals (Ismail et al. 2011). Additionally, Wang et al. showed that a high‐fat diet can significantly increase the F/B ratio in mice (Li Wang et al. 2018). Mongolian nomads adhere to a traditional dietary pattern throughout the year, primarily involving meat and dairy products, particularly in winter, fermented dairy products, and koumiss, in significant amounts. This dietary pattern essentially constitutes a high‐fat diet; thus, it is not surprising that a high F/B ratio was observed in the gut microbiome of nomads. At the genus level, *Lachnospira* was significantly enriched in the urban population. It is involved in the metabolism of various carbohydrates, particularly pectin found in fruits and vegetables, and plays a crucial role in the production of short‐chain fatty acids, such as acetic and butyric acids, in the gut (Abdugheni et al. 2022). At the species level, significant differences in the type and abundance of beneficial species were observed between the gut microbiota of the nomadic and urban populations. These findings highlight how distinct dietary structures in these regions contribute to significant variations in the gut microbiota. The levels of various bacterial species, including some beneficial species, differed between nomads and city dwellers. Nomads exhibited higher levels of opportunistic pathogens, such as *Clostridium perfringens*, in their guts. Pan demonstrated that a Western diet high in fat and protein is associated with increased levels of opportunistic pathogens (Huang and Liu 2019). Given the similarities between the nomadic and Western diets in terms of high fat and protein content, it is unsurprising that nomads also harbor more opportunistic pathogens. Corresponding to their high fat and protein intake, protein metabolites (purines and pyrimidines) were more prevalent in the gut of nomads than in urban areas.

Our investigation revealed that in Mongolia, lactic acid bacteria and *Bifidobacterium* sp. are commonly found in both urban and nomadic populations, which is noteworthy when compared to other countries or regions. Notably, despite considerable regional and seasonal variations in dietary structure, the abundance of these beneficial microbial taxa remained largely unchanged. This stability underscores the significant role of traditionally fermented dairy products, which are widely consumed in substantial quantities across the Mongolian population, in shaping and sustaining a gut environment favorable to lactic acid bacteria. Regardless of urbanization or lifestyle differences, the traditional Mongolian diet retains core features that support a microbiome enriched with lactic acid bacteria and other probiotic species. The pervasive and consistent intake of fermented dairy products appears to provide a continuous source of beneficial microbes, contributing to the maintenance of a resilient gut microbiota. Furthermore, it is plausible that the gut microbiota of the Mongolian population has undergone long‐term ecological adaptation driven by natural selection in response to the unique dietary and environmental conditions of the region. This evolutionary alignment may have resulted in a stable gut microbial community structure, wherein lactic acid bacteria establish a persistent and well‐adapted ecological niche. Consequently, these taxa exhibit limited sensitivity to dietary variations across regions and seasons, thereby maintaining their functional role in the gut microbiome. This rich assortment of lactic acid bacterial species lays the foundation for a vibrant probiotic industry in Mongolia. Gut macrobiotics can provide valuable insights into a person's health status. For example, Xu et al. demonstrated that gut microbial data combined with blood biochemical data in the second trimester could predict the risk of dyslipidemia in pregnant women during the third trimester (Yang et al. 2023). Oliver showed that the presence of specific gut microbiota could indicate the presence of metabolic features of type II diabetes (Aasmets et al. 2021). Our study found significant correlations between the bacterial species present in the guts of our Mongolian research population and various blood parameters, including glucose, lipids, and liver and kidney function tests, as well as the intake of energy, protein, and carbohydrates.

It is important to acknowledge the significant limitations of this study. First, cultural sensitivities surrounding personal information led to a considerable proportion of participants withholding key demographic data, such as age and body mass index (BMI). This limitation restricted our capacity to control for potential confounding variables, thereby influencing the interpretation of our findings. Second, although this study successfully identified meaningful associations, further research is warranted to elucidate the underlying mechanisms through which the gut microbiota influences human health. Such inquiries will require more granular methodological approaches, including refined population stratification and advanced sequencing technologies. To mitigate these limitations, future investigations should aim to recruit a more geographically and demographically diverse cohort across Mongolia, emphasizing the inclusion of participants amenable to providing comprehensive baseline data. Additionally, longitudinal cohort designs should be employed to monitor gut microbiome trajectories in populations transitioning from nomadic to urban lifestyles. Integrating multi‐omics analyses, including metagenomics, metabolomics, and transcriptomics, is crucial for systematically exploring microbial adaptations to environmental shifts. This holistic approach is essential for disentangling the complex interactions between dietary patterns, microbial composition, and host health outcomes.

## 5 Conclusions

This cross‐sectional study revealed significant differences in dietary nutrition, gut microbiota, and health status among the geographically diverse Mongolian populations. The correlations between gut microbiota, nutrient intake, and blood indices in Mongolians provide novel evidence for the regulation of gut microbiota and health status through dietary intervention. Nevertheless, cultural norms regarding personal privacy constrained the acquisition of comprehensive demographic data, limiting our ability to control for potential confounding variables. Furthermore, although we established several key associations, the underlying biological mechanisms through which the gut microbiome influences host health remain to be fully elucidated. Despite these limitations, our findings contribute valuable insights to the growing body of literature on host–microbiota interactions and underscore the importance of culturally contextualized diet‐based strategies for promoting public health. Future research incorporating longitudinal designs and integrative multi‐omics approaches will be essential for advancing our mechanistic understanding and translating these insights into effective, personalized health interventions.

## Author Contributions

**Bilige Mengheand Davaasambuu Ganmaa:** conceived the study, designed the study, protocol development. **Zhixin Zhao:** data curation (equal), formal analysis (equal), writing‐original draft (equal). **Battogtokh Chimeddorj, Enkhtsetseg Tserenkhuu, Munkhtsetseg Ochirdanzan, Dulamsuren Ganpurev, Enkhmaa Davaasambuu, Yagaantsetseg Talkhaa, Yansanjav Narankhuu, Sabri Bromage and Christina Warinner:** recruited volunteers, collected samples. **Zhihong Sun, Wenjun Liu, Feiyan Zhao, Weicheng Li and Wusigale:** supervised all experiments. **Shuying Yang, Mengdi Zhang and Weng Fun:** drafted the statistical analysis plan. All authors have discussed the results and commented on the manuscript. All authors provided substantive comments and approved the final version for submission.

## Consent

The authors have nothing to report.

## Conflicts of Interest

The authors declare no conflicts of interest.

## Supporting information


**Figure S1** Significantly different relative abundance at the family, genus, and species levels between summer and winter in urban populations. The horizontal line inside the box represents the median and each dot represents the data point of a participant. Significant differences between different groups are represented by * (*p* < 0.05), ** (*p* < 0.01). Yellow and blue colors represent the UW and US, respectively. UW and US represent urban winter and summer, respectively.

**Table S1‐S6**: Supplementary Tables.

## Data Availability

The raw sequence data reported in this paper have been deposited in the Genome Sequence Archive (Genomics, Proteomics & Bioinformatics 2021) in National Genomics Data Center (Nucleic Acids Res 2022), China National Center for Bioinformation/Beijing Institute of Genomics, Chinese Academy of Sciences (GSA: CRA015216) that are publicly accessible at https://ngdc.cncb.ac.cn/gsa.
