# Supplementary material for: Dietary Nutrition, Gut Microbiota, and Health Status Across Geographically Diverse Populations in Mongolia: A Cross‐Sectional Study
Source: Food Sci Nutr. 2025 Jul 4;13(7):e70531. doi: 10.1002/fsn3.70531 (PMC12227796; doi:10.1002/fsn3.70531)
Supplement: Supplementary file 1 — Figure S1 Significantly different relative abundance at the family, genus, and species levels between summer and winter in urban populations. The horizontal line inside the box represents the median and each dot represents the data point of a participant. Significant differences between different groups are represented by * (p < 0.05), ** (p < 0.01). Yellow and blue colors represent the UW and US, respectively. UW and US represent urban winter and summer, respectively. [file FSN3-13-e70531-s002.docx]

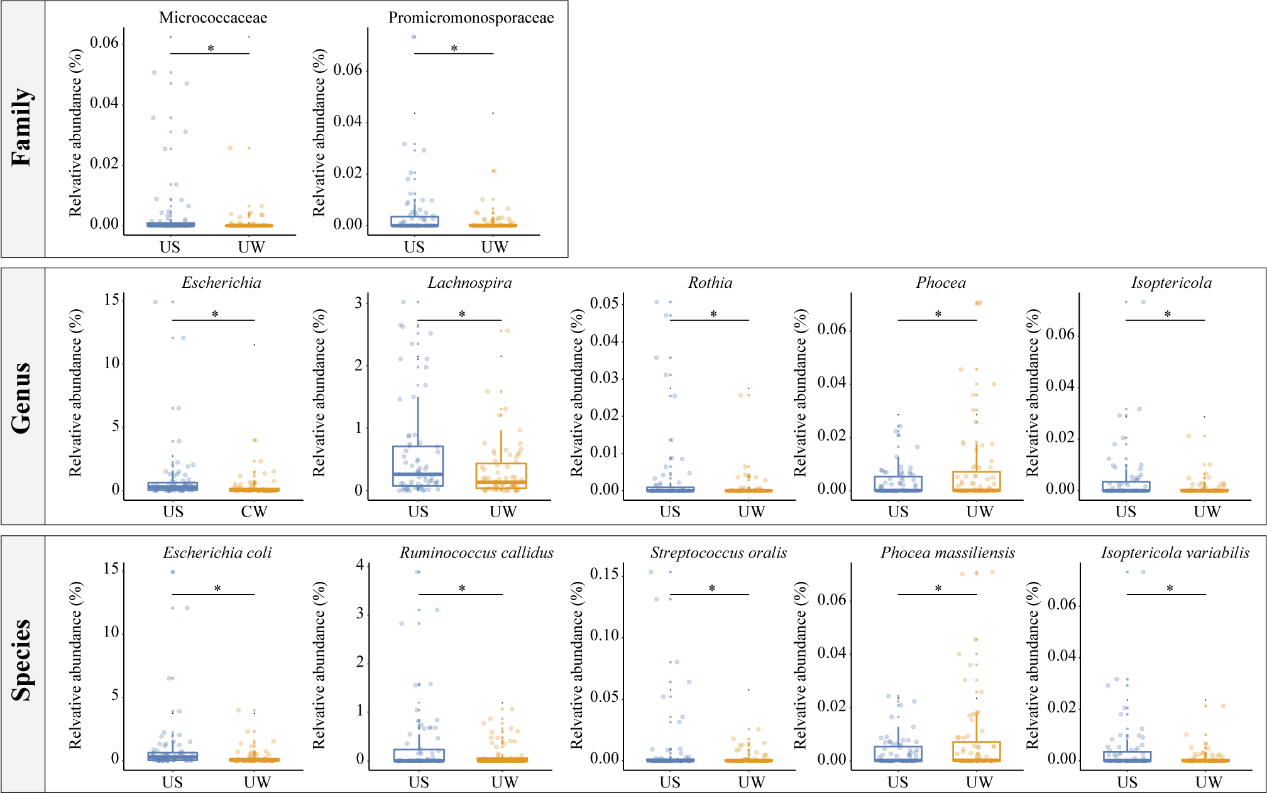


**Figure S1.** Significantly different relative abundance at the family, genus, and species levels between summer and winter in urban populations. The horizontal line inside the box represents the median and each dot represents the data point of a participant. Significant differences between different groups are represented by * (*P* < 0.05), ** (*P* < 0.01). Yellow and blue colors represent the UW and US, respectively. UW and US represent urban winter and summer, respectively.
